# Gridded mobile source emissions with multiple processes and pollutants from 2011 to 2020 in China

**DOI:** 10.1038/s41597-025-05690-6

**Published:** 2025-08-01

**Authors:** Junchao Zhao, Zhaofeng Lv, Zhenyu Luo, Zhining Zhang, Haitong Zhe Sun, Wenxin Cao, Wen Yi, Yongyue Wang, Hezhong Tian, Yan Ding, Kebin He, Huan Liu

**Affiliations:** 1 State Key Laboratory of Regional Environment and Sustainability, School of Environment, Tsinghua University, Beijing, 100084 China; 2 Key Laboratory of Vehicle Emission Control and Simulation of Ministry of Ecology and Environment, Vehicle Emission Control Center, Chinese Research Academy of Environmental Sciences, Beijing, 100012 China; 3 Centre for Sustainable Medicine (CoSM), National University of Singapore, Singapore, 119228 Singapore; 4 State Key Joint Laboratory of Environmental Simulation & Pollution Control, School of Environment, Beijing Normal University, Beijing, 100875 China

**Keywords:** Environmental impact, Atmospheric chemistry

## Abstract

Accurate and comprehensive estimation of mobile source (MS) emissions is essential for air quality management and atmospheric research. However, existing emission inventories often lack sufficient coverage of MS categories, emerging pollutants, and newly developed model parameterizations. Here, we present the Gridded Mobile-source Emission Dataset (GMED), a model-ready emission inventory with detailed source classification and multi-pollutant coverage. GMED provides monthly emissions from 2011 to 2020 at a spatial resolution of 36 km × 36 km, covering eight MS categories and including both tailpipe and non-exhaust emissions. It incorporates several methodological improvements and integrates localized measurements from multiple sources, including emission factors, organic compound speciation, spatiotemporal proxies, and activity data. Validation involves comparisons with established inventories and cross-checks of key model parameters, both showing good agreement across multiple metrics. GMED outputs also align well with high-resolution emission inventories derived from big traffic data. As a validated, long-term dataset, GMED supports emission assessments, air quality modelling, and policy evaluation, helping to fill a critical gap in differentiated, multi-year MS emission inventories.

## Background & Summary

With rapid economic development and growing transportation demand, mobile sources (MSs) have become one of the most significant contributors to urban air pollution^1,2^. The growing number and diversity of MSs, ranging from on-road vehicles to ships, aircraft, and non-road machinery (NRM), have substantially intensified their emissions in many regions. In China, for instance, the number of motor vehicles surpassed 400 million by the end of 2023, underscoring the accelerated growth in MS ownerships^3^. To address the escalating environmental pressures, a series of control policies have been introduced and updated to mitigate MS emissions. In addition, numerous studies have assessed the effectiveness of these control strategies across regions and MS categories, providing scientific support for policy refinement^4,5^. However, due to the complex categorization and dynamic behaviour of MSs, quantifying their emissions with sufficient accuracy remains a major challenge^6^. Accurate and representative emission inventories provide the essential scientific basis for air quality modelling and the formulation of effective, source-specific control strategies.

Recent advances in emission measurements have highlighted substantial differences in chemical composition, emission intensities, operating conditions, and spatiotemporal patterns across MSs, both between domestic and international fleets and among different MS subcategories^7–9^. These findings underscore the growing need for source-differentiated and localized MS emission inventories that capture regional differences in fleet composition, activity patterns, and emission characteristics. In addition, most currently available emission inventories still aggregate all MSs into a single, undifferentiated category, without a flexible framework to enable source-specific characterization^10^. This simplification hampers the integration of refined modules for specific MS types and limits the capacity for targeted scenario simulations, leading to less flexible inventories and increased uncertainty in policy evaluation^11^.

Beyond the lack of source differentiation, recent shifts in emission control focus have further expanded the scope and complexity of emission inventory demands. With tailpipe emissions dramatically regulated, the relative importance of non-tailpipe sources, such as evaporative emissions and tire or brake wear, has grown dramatically^12,13^. At the same time, emerging pollutants, like ammonia (NH_3_) and the full-volatility organic compounds, have been identified through advanced emission measurements, further expanding the scope of emission inventory demands for various MSs^14,15^. Existing systems often lag in integrating such developments, lacking parameterizations for estimating new pollutants, emission processes, or inter-annual parameter variability. These growing complexities underscore the need for basic and classified emission inventories that can accurately represent source-specific emission characteristics^16^.

To address the limitations of existing emission, it is crucial to develop detailed emission inventory algorithm that align with varying data conditions and policy needs. Emission inventory technologies differ substantially across MS categories, reflecting disparities in data availability and technological feasibility. For on-road vehicles, ships, and aircraft, the increasing integration of real-time traffic data has enabled high-resolution, near-real-time emission estimations^17–20^. However, many other MS types still rely primarily on top-down approaches based on aggregated statistic data^21^. Nevertheless, each emission inventory approach serves distinct analytical purposes^8^. For instance, near-real-time emission inventories are well-suited for evaluating short-term control measures, localized pollution episodes, and diurnal variations. But their heavy reliance on high-resolution real-time activity data restricts both scalability and historical retrospective analyses, particularly where data are scarce^18^. By contrast, regional-scale inventories derived from statistical data are easier to implement across broader regions and longer timeframes. Their consistent temporal coverage makes them especially useful for tracking long-term emission trends and evaluating how control strategies have performed over time^16^. Therefore, improving the representativeness of top-down inventory methodologies is essential for generating more accurate, multi-year emission inventories that can accurately capture the evolving patterns of MS emissions.

Despite progress in measurements and emission inventory models, improvements have often been confined to specific components, while other modules remain overly simplified. Common issues for MS emission inventories include delayed incorporation of updated emission data, limited coverage of pollutants and emission processes, and a lack of algorithms to reflect inter-annual variability in key parameters^16,22^. These fragmented improvements reduce the overall consistency and reliability of emission inventories. To address these challenges, a more comprehensive framework is needed, allowing for the consistent integration of diverse data sources and iterative updates.

In this context, this study developed a source-classified model-ready MS emission inventory covering the period from 2011 to 2020. This dataset integrates multi-process emission estimations across a wide range of pollutants, including both regulated and emerging species, as well as evaporative and wear emissions. It also provides year-by-year emission maps at a gridded spatial resolution of 36 km × 36 km. The inventory framework is compiled using R language. This allows for the integration of updated emission factors, activity data, correlations for various emission inventory modules, chemical compositions for organic compounds, and multi-source spatial proxies. For each MS category, the modelling approach is adapted to the available activity data. By incorporating various improvements from literatures and our previous work, this model is well-suited for applications in scenario analysis, air quality modelling, and policy development, particularly in regions where differentiated mobile source data and long-term emission baselines have been lacking.

## Methods

**Fig. 1:**
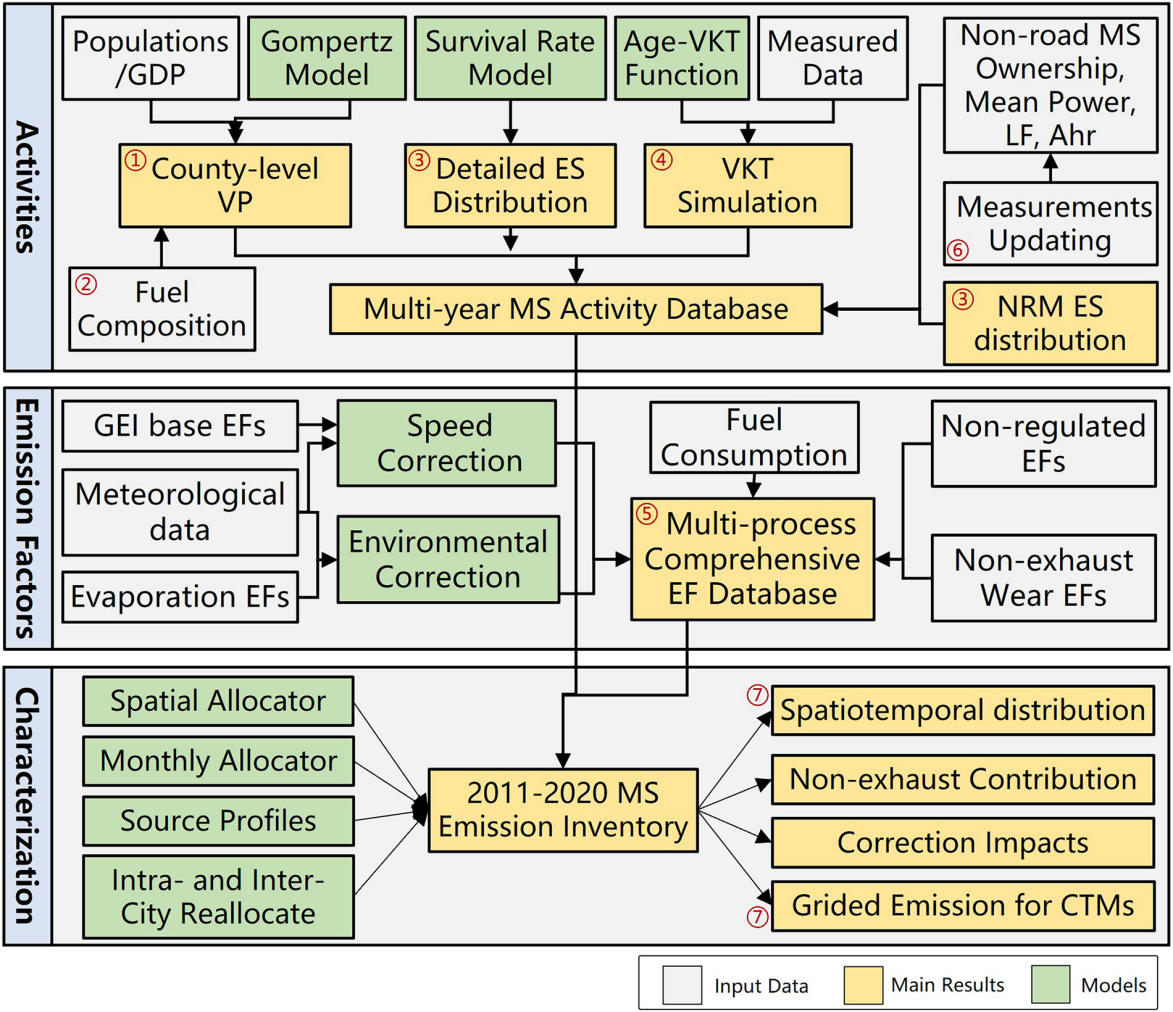
Diagram for emission estimation methods. MS: Mobile Source; LF: Load Factors; Ahr: Annual Activity Hours; ES: Emission Standards; VP: Vehicle Populations; Age: Vehicle Age: NRM: Non-road Machinery; GEI: Technical Guidelines on Emission Inventory; EFs: Emission Factors; CTMs: Chemical Transportation Models; ①-⑦ represents the improvements of GMED, including modelling VP for each county, reallocation the fuel compositions specific to each province, modelling the ES distribution and VKT variations, compiling a comprehensive EF database for MS, integrating measured activity data for non-road MS, and providing detailed spatiotemporal allocation of emissions.

The Gridded Mobile-source Emission Dataset (GMED) with multi-pollutant is developed through the integration of statistical data, experimental measurements, alongside various peer-reviewed correction algorithms. This integration enables further optimization of key parameters within the emission estimation framework, including ownership distributions across MS categories, vehicle kilometres travelled (VKT), emission standard (ES) distributions, emission factors (EFs), and, for non-road machinery (NRMs), load factors (LFs) and annual activity hours (Ahr). This model also facilitates the spatiotemporal distribution of emissions and the chemical speciation of organic pollutants. In addition, the modular and programmatic design of GMED enables flexible application in various scenarios, such as emission forecasting and policy evaluation. The integrated framework for emission estimation methods is illustrated in Fig. 1.

### Basic methodologies for GMED

For the vehicular module, emissions are estimated for all 2,848 counties across mainland China (as defined by the 2022 administrative divisions, excluding Hong Kong, Macau, and Taiwan), covering tailpipe, evaporative, and non-exhaust emissions such as brake and tire wear. The methodology is expressed as Eqs. 1–4^23–25^:

1 $${E}_{\text{T},\text{p}}={\sum }_{\text{i},\text{j},\text{f},\text{m}}\left({\text{VP}}_{\text{i},\text{j},\text{f}}\times {\text{ES}}_{\text{i},\text{j},\text{f}}\times {\text{VKT}}_{\text{i},\text{j},\text{f},\text{m}}\times {\text{EF}}_{\text{i},\text{j},\text{f}}\right)$$

2 $${E}_{\text{E},\text{p}}={\sum }_{\text{k},\text{j},\text{m}}\left({\text{VP}}_{\text{j}}\times {\text{ES}}_{\text{j}}\times {\text{EF}}_{\text{j},\text{k}}\right)$$

3 $${E}_{\text{W},\text{p}}={\sum }_{\text{i},\text{r}}\left({\text{VP}}_{\text{i}}\times {\text{VKT}}_{\text{i},\text{r}}\times {\text{EFW}}_{\text{j},\text{k}}\right)$$

4 $${E}_{{\rm{CO}}2}={\sum }_{{\rm{i}},{\rm{f}}}\left({{\rm{VP}}}_{{\rm{i}},{\rm{f}}}\times {{\rm{VKT}}}_{{\rm{i}}}\times {{\rm{FC}}}_{{\rm{i}},{\rm{f}}}\times {{\rm{\rho }}}_{{\rm{f}}}\times {{\rm{NCV}}}_{{\rm{f}}}\times {{\rm{EF}}}_{{\rm{f}}}\times {{\rm{O}}}_{{\rm{f}}}\right)$$

where *E*_*T*,*p*_, represents the tailpipe exhaust of pollutant *p*; VP represents the vehicle population; ES stands for emission standard; VKT denotes vehicle kilometres travelled (km); EF represents emission factor; Pollutant p in exhaust emissions contain CO, HC, NOx, PM_2.5_, PM_10_, VOCs, IVOCs, SVOCs, xLVOCs and NH_3_ (mg/km); *i* denotes the vehicle type, including Light-duty passenger vehicle (LDV), Medium-duty passenger vehicle (MDV), Heavy-duty passenger vehicle (HDV), Light-duty truck (LDT), Medium-duty truck (MDT) and Heavy-duty Truck (HDT), Motorcycle emissions are included based on the emission inventory from our previous research^26^; *j* denotes ES, including Pre-China I to China VI; *f* represents fuel types, considering gasoline, diesel, other fuels (natural gas vehicles) and electric vehicles (only have wear emissions). *m* denotes different months; *E*_*E*,*p*_, and *E*_*W*,*p*_ represent the evaporative and wear emissions of pollutant *p*, respectively; *k* represents different evaporative emission processes of gasoline vehicles, including refuelling, hot soak, permeation, diurnal and running loss; EFW denotes the wear emission factor; *r* denotes road types, including urban roads, rural roads, and highways; *E*_CO2_ denotes vehicular CO_2_ emissions; FC denotes the fuel consumptions (L/100 km); *ρ* denotes fuel density (g/L); NCV denotes Net Calorific Value; EFC denotes the Carbon emission factor; *O* denotes the Carbon Oxidation Rate.

For non-road MS modules, emissions are quantified independently and categorized into four subgroups: non-road machinery (NRM), aircraft, railway internal combustion engines (RE), and ships. In this study, the methodology for NRM emission inventory is expressed as Eq. 5^27^. RE emission is calculated based on fuel consumption method (Eq. 6), and only aircraft (passenger and cargo aircraft) emissions during Landing and Take-Off (LTO) cycle are considered^27^ (Eq. 7). Based on Automatic Identification System (AIS) big data, muti-year shipping emission within 200 nautical miles are estimated by shipping emission model (SEIM), which is a bottom-up model that incorporates detailed vessel activity data, including real-time vessel positions, speeds, and technical parameters of ocean-going, coastal and river vessels^28,29^.

5 $${E}_{\text{NRM},\text{p}}={\sum }_{\text{i},\text{j},\text{k}}({\text{P}}_{\text{i},\text{j},\text{k}}\times {\text{G}}_{\text{i},\text{j},\text{k}}\times {\text{LF}}_{\text{i},\text{j},\text{k}}\times {\text{Ahr}}_{\text{i},\text{j},\text{k}}\times {\text{EFG}}_{\text{i},\text{j},\text{k}})$$

6 $${E}_{\text{RE},\text{p}}=\sum Y\times {\text{EF}}_{\text{p}}$$

7 $${E}_{\text{LTO},\text{p}}=\sum {N}_{\text{LTO}}\times {\text{EF}}_{\text{LTO},\text{p}}$$

where *E*_NRM,p_ represents the NRM emission of pollutant *p*; *P* represents the ownership of NRM; *i* denotes the NRM type, including construction machinery (NRCM), agricultural machinery (NRAM), and small general-purpose machinery (SGPM). Furthermore, NRCM contains excavators, bulldozers, loaders, forklifts, road rollers, pavers, graders, and others. NRAM comprises large and medium-sized tractors, small tractors, combine harvesters, irrigation and drainage machinery, and others. SGPM encompasses both handheld and non-handheld devices; *j* denotes ES, including Pre-China I to China III; *k* denotes power distributions, including below 37 kW, 37~75 kW, 75~130 kW, and above 130 kW; EFG stands for power-based EF. *G* denotes the average rated net power; LF stands for the load factor; hr represents the annual operating hours; *E*_RE,p_ represents the RE emission of pollutant *p*; *Y* stands for the fuel consumption; EF stands for emission factor; E_LTO,P_ represents the aircraft emissions during LTO cycle; *N*_LTO_ denotes the number of LTO cycles; EF_LTO,P_ represents the LTO-based emission factor for pollutant *p*.

### Input datasets and the improvements of GMED

Most existing improvements to emission inventory models primarily focus on evaluating relative emission changes before and after specific modifications. However, other components of these models are often overly simplified, resulting in an incomplete evaluation result. To overcome this limitation, this study integrates a broader range of improvements for MS emissions, aiming to develop a more representative emission inventory for MS, which can be easily incorporated into chemical transport models (CTMs). The seven key improvements implemented in the newly developed GMED framework are shown in Fig. 1, and detailed in the following sections.

### Modelling the vehicle population for each county

This study used the Gompertz model to simulate the VP in each county. The Gompertz model is a widely used sigmoidal growth function that effectively captures the S-shaped trajectory of VP growth as economies develop^30^. In this study, the model establishes a nonlinear relationship among VP, population, and *G* (per-capita GDP), which enables the downscaling of provincial-level VP estimates to the county level with improved spatial resolution^30^. The mathematical representation of the Gompertz model can be found in Eqs. 8–9^30^.

8 $$\text{VPK}=\text{SVPK}\times {\text{e}}^{\alpha {e}^{\beta G}}$$

9 $$\mathrm{ln}\left(-\mathrm{ln}\left(\frac{{\text{VPK}}_{\text{c}}}{\text{SVPK}}\right)\right)=\mathrm{ln}\left(-{\alpha }_{c}\right)+{\beta }_{c}{G}_{c}$$

where *c* represents different cities; VPK denotes the VP per thousand people; SVPK represents the saturation value of VPK, conventionally set to 500 as per prior research. *α* and *β* are key parameters of the Gompertz model; *G* denotes per-capita GDP.

Gompertz model requires the key parameters (*α* and *β*) for each region. As shown in Eq. 8, there is a linear correlation between ln(−ln(VPK/SVPK)) and *G*. This study uses statistical data from 2011 to 2020 to derive region-specific α and β values based on the methods described in Eqs. 10–12 (https://data.stats.gov.cn/search.htm?s=GDP). Given the intra-city consistency in economic development, population, and vehicle ownership patterns, the parameter *α* is assumed to be uniform across all counties within a city (Eq. 10), while *β* is allowed to vary among counties to reflect local heterogeneity (Eqs. 11, 12). Therefore, the β values are scaled at the county level by constraining each county’s per-capita GDP within the upper and lower bounds defined by its corresponding city (Eq. 12)^30^.

10 $${\alpha }_{{\rm{c}},{\rm{d}}}={\alpha }_{{\rm{c}}}$$

11 $${\beta }_{\text{c},\text{d}}={{SF}}_{\text{c},\text{d}}{\beta }_{\text{c}}$$

12 $${\text{SF}}_{\text{c},\text{d}}=\left\{\begin{array}{c}\frac{{G}_{\text{c},\min }}{{G}_{\text{c},\text{d}}},{G}_{\text{c},\text{d}}\le {G}_{\text{c},\min }\\ 1,{G}_{\text{c},\min } < {G}_{\text{c},\text{d}}\le {G}_{\text{c},\max }\\ \frac{{G}_{\text{c},\max }}{{G}_{\text{c},\text{d}}},{G}_{\text{c},\text{d}} > {G}_{\text{c},\max }\end{array}\right.$$

13 $$\mathrm{ln}\left(-\mathrm{ln}\left(\frac{{\text{VPK}}_{\text{c},\text{d}}}{\text{SVPK}}\right)\right)=\mathrm{ln}\left(-{\alpha }_{\text{c},\text{d}}\right)+{\beta }_{\text{c},\text{d}}{G}_{\text{c},\text{d}}$$

where *c* represents different cities; *d* represents the counties within city *c*; SF represents the scale factors; *G*_*c*,min_ and *G*_*c*,max_ stand for the minimum and maximum values of *G* in city c during 2011–2020, respectively; VPK and SVPK are same with Eq. 8.

Gompertz model fitting (Eqs. 8, 9) was conducted at the city level using historical VPK and *G* data, yielding city-specific Gompertz parameters (*α* and *β*). These parameters were then adjusted (Eqs. 10–12) to derive corresponding *α* and *β* values for each county, enabling the calculation of county-level SVPK (Eq. 13). In cases where the model fitting at the city level produced a low correlation (*R*^2^ < 0.5), provincial-level Gompertz parameters were used instead. Based on the estimated SVPK and county-level population data, estimated county-level VP were derived. Finally, the total provincial VP was allocated to individual counties in proportion to their estimated county-level VP values^30^.

The statistical data required for the Gompertz model includes annual records of permanent population and GDP for each province, city and county, as well as data on VP for each province and city. This study established a comprehensive database for permanent population, GDP and VP at various administrative levels by consolidating information from various statistical yearbooks and bulletins, including provincial/municipal statistical yearbooks (https://data.stats.gov.cn/easyquery.htm?cn=E0103). Relevant data can be retrieved using the Chinese keywords for “population”, “GDP”, and “vehicle population”. Due to variations in statistical methodologies and data availability across regions and years, missing values in the compiled dataset were filled using linear interpolation. The administrative divisions used for the multi-year emission inventory is based on the 2022 administrative boundary status.

### Reallocation of the fuel compositions specific to each province

This study integrated VP data from the 2021 vehicle traffic accident compulsory insurance (TACI) system (the earliest year such data are available, https://www.daas-auto.com/supermarket_data_De/727.html). The TACI dataset includes 264 million records covering all vehicle types across all provinces. Fuel types were categorized as gasoline, diesel, natural gas, and electricity. The total number of electric vehicles (EVs) is derived from public data information from the Ministry of Public Security (https://www.mps.gov.cn/n2254314/n6409334/n9194010/c9217870/content.html). To exclude EVs from the total VP, the total number of EVs from for the period 2011–2020 was allocated to each province based on the relative provincial shares in the 2021 TACI dataset. The allocated EV ownership was then subtracted from the total VP in each province. Finally, the relative shares of three fossil fuel types, gasoline, diesel, and natural gas, derived from the 2021 TACI data were used to allocate the adjusted multi-year vehicle population (excluding EVs) in each province.

This study further examined interannual variations in fuel composition, with a specific focus on natural gas HDVs. Annual total VP of natural gas HDV for 2011–2020 was obtained from the Transportation Industry Development Statistical Bulletin (https://xxgk.mot.gov.cn/2020/jigou/zhghs/202105/t20210517_3593412.html). These national total amounts were then distributed to individual provinces according to the provincial shares derived from the TACI data. The remaining HDVs (total VP minus the VP from natural gas HDVs) were further disaggregated into gasoline and diesel categories using their relative proportions from the TACI dataset. This detailed fuel composition enabled the identification of regions with relatively high shares of natural gas HDVs. For example, following the implementation of China VI emission standards, natural gas vehicles were widely promoted in provinces like Shanxi, a major hub for bulk goods transport, due to their lower after-treatment upgrade costs. Consistent with this trend, our results show that Shanxi had the highest proportion of natural gas HDVs, reaching 36% (Fig. S1). Moreover, interprovincial differences in fuel composition reached up to 50%, underscoring the importance of incorporating region- and vehicle-specific fuel breakdowns in emission inventory models.

### Modelling emission standard distribution

**Fig. 2:**
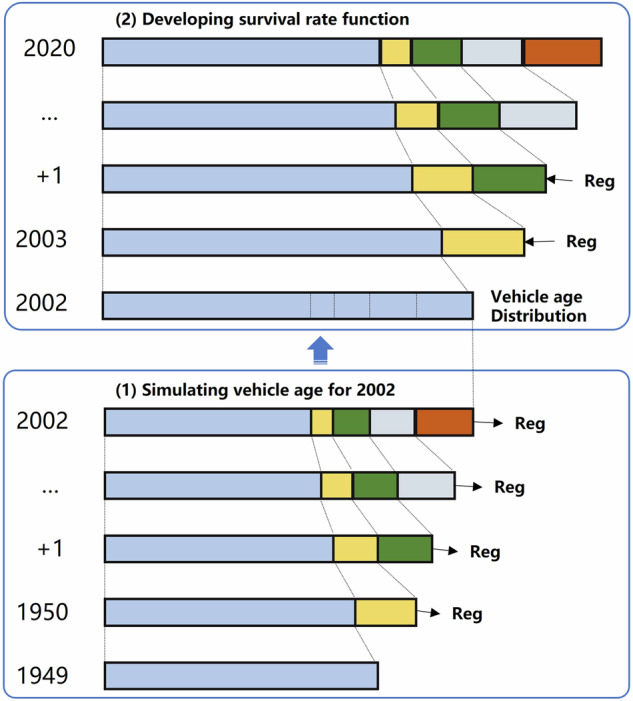
Technical framework of the survival rate function for vehicle age simulation. Reg: newly registered VP.

The survival rate function defines the relationship among three key variables: total VP, newly registered VP, and scrappage rate. Given any two of these parameters, the third can be calculated iteratively (Fig. 2)^30^. This function is used to simulate the age distribution of different vehicle fleets (Eqs. 14–16). Assuming vehicles follow the emission standards in place at the time of registration, the ES distribution is derived by mapping the age structure to the respective ES implementation schedules across fuel types, provinces, and vehicle categories.

The year 2002 was selected because it marks the point from which both key datasets (new registrations and total VP) are consistently available. For the first step, by using the scrappage rate from literatures^30^, we intend to get an average age distribution of VP for 2002. To improve the accuracy of simulated age distributions, this study initiated the iterative process from the earliest available statistical year for each province (https://data.stats.gov.cn/easyquery.htm?cn=E0103). Among the collected data, 20 provinces have historical records dating back to 1949, 3 provinces to 1961, 5 provinces to 1978, and 2 provinces to 1985. Before 1991, only total VP data were available. Thus, an average age distribution was fitted for all vehicle types before 1991, by using the default survival curve parameters from literature^30^. Between 1991 and 2002, the averaged age distributions were then divided into passenger and truck vehicles using Eqs. 14–16. Then, a basic average age distribution of passenger and truck vehicles for 2002 VP were achieved. From 2002 onward, next simulation would model more accurate scrappage rate by using the statical data of newly registered VP, total VP, along with detailed age distribution data in 2002 from the previous step. This enabled the improvement of the survival rate function parameters *T* and *b* by minimizing the deviation between simulated and statistical VPs. The optimized parameters (listed in Table S1) produced refined age distributions and improved survival curves for each vehicle subgroup. Through this multi-stage calibration process, a more accurate survival rate function was established, allowing for robust simulation of vehicle age distributions and model years across regions and years.

This study adopted the ES implementation timelines reported in the China Mobile Source Environmental Management Annual Report^3^. Furthermore, specific implementation timelines were delineated for regions such as Beijing, Shanghai, and Guangdong, where the emission control measures exceed the national average level^31^. Additionally, discrepancies were also considered for provinces and municipalities such as Beijing, Shanghai, Tianjin, Hebei, Shandong, Henan, Hainan, and Guangdong, which initiated the early implementation of China VI ES (https://www.gov.cn/xinwen/2019-07/01/content_5405040.htm). This approach fully reflects the variations in the implementation timing of vehicle ES across different vehicle types and fuel types. The simulation method of NRM ES is similar to that of vehicle ES, categorized based on the annual newly registered population and the implementation date of emission standards.

14 $${S}_{\text{r},\text{y}-\text{r}}=\exp \left[-{\left(\frac{\left(y-r\right)+b}{T}\right)}^{b}\right]$$

15 $${\text{VP}}_{y}=\mathop{\sum }\limits_{r=1949}^{y}({\text{Reg}}_{r}\times {S}_{r,y-r})$$

16 $${\text{Reg}}_{y+1}={\text{VP}}_{y+1}-{\text{VP}}_{y}$$

where *r* represents the vehicle registration year; *y* denotes the year of emission inventory; *y - r* stands for the vehicle age; *S* represents the survival rate of vehicle at different age; *T* and *b* are key parameters of the survival rate function for different vehicle types; Reg denotes the new registered VP; VP_*y*_ indicates the ownership of new registered vehicles retained until the emission inventory year *y*.

### Modelling annual variations of VKT

Vehicle usage typically declines with age, yet this effect is often overlooked in vehicle kilometre travelled (VKT) estimations. In this study, we used the ES-based VKT for 2015, which was directly derived from previous analyses based on over 70,000 GPS signal samples and 2 million trajectory records collected via GPS or BeiDou systems across 30 provinces^32,33^. Based on these data, an age-VKT function was employed to allocate VKT across vehicle age groups and road types^31,34^.

For passenger vehicles, the dataset includes detailed classifications by province, fuel type, vehicle category, and age. First, the weighted average vehicle age (M) for each category and province was calculated using Eq. 17. Then, the relative change in fitted VKT for other years (2011–2014 and 2016–2020) was estimated relative to 2015 using the age-VKT function (Table S2). These changes were then applied to the 2015 baseline to derive annual VKT values (Eq. 18). For trucks, the 2015 data are categorized by province, fuel, vehicle type, and emission standard (ES). To estimate VKT for other years, we used ES as a bridge between vehicle age and VKT. Specifically, vehicle age distributions were first mapped to ES groups using survival curves (Eqs. 14–16), enabling the reconstruction of an age–VKT relationship for trucks. This reconstructed function was then used to estimate VKT for other years. The overall decreasing trends in VKT with newer inventory years (corresponding to a lower average vehicle age) were presented in Fig. S2.

17 $${M}_{a,\text{i},\text{j}}={\sum }_{n}({\text{VP}}_{a,\text{i},\text{j},n}\times n)/{\text{VP}}_{a,\text{i},\text{j}}$$

18 $${\text{VKT}}_{a,\text{i},\text{j}}={\text{f}}_{\left({M}_{a,\text{i},\text{j}}\right)}/{\text{f}}_{\left({M}_{2015,\text{i},\text{j}}\right)}\times {\text{VKT}}_{\text{base},\text{i}}$$

where *M* represents the weighted average vehicle age; *a* denotes the inventory years; *i* represents the vehicle types; *j* denotes the ES; VP represents the vehicle population; *n* represents the vehicle age for certain subsets; *f*(*M*) stands for the fitted VKT from age-VKT function (Table S2); VKT_base,i_ represents the measured VKT of vehicle type *i* for 2015.

### Comprehensive EF database for MS

A comprehensive MS EF database was compiled by integrating the measured EF data^22,35–46^, chemical compositions of organic pollutants^47–50^, GEI for on-road and non-road MS^23,27,51,52^, and EF correlation algorithms^24,25^. This database characterizes the EFs of all types of MS, as well as different emission process (Eqs. 1–3), more description could be found in Table S3. For regulated pollutants (CO, HC, NOx, PM_2.5,_ and PM_10_) from on-road vehicles, the correlation methods (Eqs. 19–22) account for the impact from various factors including meteorological conditions (such as temperature, humidity and atmospheric pressure), geographical factors (altitude), driving conditions (such as average speeds on different roads), fuel quality, and deterioration.

19 $${\text{EF}}_{\text{p},\text{i},\text{j},\text{f},m,\text{k},\text{r}}={\text{BEF}}_{\text{p},\text{i},\text{j},\text{f}}\times {\varphi }_{\text{p},\text{i},\text{f},m,\text{k}}\times {\omega }_{\text{p},\text{i},\text{f},\text{k}}\times {\theta }_{\text{f},m,\text{k}}\times {\tau }_{\text{r}}\times {\xi }_{\text{p},\text{i},\text{j},\text{f}}$$

20 $${{\rm{\theta }}}_{{\rm{f}},m,{\rm{k}}}=1.0-\left(\left(\max \left(\min \left({{\rm{SH}}}_{m,{\rm{k}}},124\right),21\right)-75\right)\times {C}_{{\rm{f}}}\right)$$

21 $${f}_{\left(V\right)}=\frac{\left({\rm{\alpha }}\times {V}^{2}+{\rm{\beta }}\times V+{\rm{\gamma }}+\delta /V\right)}{\left(\varepsilon \times {V}^{2}+{\rm{\zeta }}\times V+\eta \right)}\times \left(1-{\rm{RF}}\right)$$

22 $${\tau }_{\text{r}}={f}_{(V={V}_{\text{r}})}/{f}_{(V=30)}$$

where BEF represents the base EFs in GEI; *p* denotes different regulated pollutants; *i*, *j*, *f*, *m*, *k* and *r* represent vehicle types, ES, fuel types, months, provinces and road types, respectively; *φ* and *ω* represent temperature correction factor and altitude correction factor, both values are derived from the recommended values in GEI^23^. *θ* represents humidity correction factor, and the correction algorithm (Eq. 20) for NOx EFs was referenced from MOVES2014 model^24^; *τ* represents the speed correction factor, and ${f}_{(V)}$ is utilized to fit speed-corrected EFs based on the COPERT model^25^. As the BEF provided in the GEI represents the EFs under the baseline driving conditions (speed of 30 km/h), Eqs. 21–22 are employed to calculate the speed-corrected EFs under varying road grades with different average driving speeds. The average driving speeds of different vehicle types on different road grades are gathered from literature^31^; Additionally, the effects of vehicle deterioration and fuel sulfur content on EFs are referenced from the GEI and are represented as *ξ* in this study^23^. In Eq. 20, SH represents specific humidity, which are calculated based on the temperature, relative humidity, and atmospheric pressure (https://www.ncei.noaa.gov/data/global-summary-of-the-day/archive/). The historical monthly meteorological observations of environmental temperature, humidity, and atmospheric pressure are derived from NOAA (National Oceanic and Atmospheric Administration) data (https://www.ncei.noaa.gov/data/global-summary-of-the-day/archive/); *C* represents the empirical coefficient, with a value of 0.0038 for gasoline and natural gas vehicles, while 0.0026 for diesel vehicles^24^; *α*, *β*, *γ*, *δ*, *ε*, *ζ*, *η*, and RF represent empirical parameters^25^.

For organic compounds EFs, this study categorizes full-volatility organic compounds into four groups based on their saturation vapor concentration (C*): VOCs (C* > 3 × 10^6^ μg/m^3^), IVOCs (300 μg/m^3^ < C* < 3 × 10^6^ μg/m^3^), SVOCs (0.3 μg/m^3^ < C* < 300 μg/m^3^), and xLVOCs (organic compounds with even lower volatility)^53^. Additionally, it should be noted that HC emissions were used solely for validation purposes in this study; the dataset itself reports total organic compound emissions instead. The IVOC EF database, developed in our previous work across various MS types^22^, is adopted in this study. EFs for SVOCs and xLVOCs are estimated using the integration method proposed by Chang *et al*., based on primary organic aerosol EFs and corresponding source profiles^48,50^. These organic EFs (including xLVOCs, SVOCs, and IVOCs) represent the total contributions from both gaseous and particulate phases. Furthermore, VOCs are sourced from GEI-recommended values and literature data^35,51^. In addition, evaporative VOC EFs from gasoline vehicles, including refuelling, hot soak, permeation, diurnal, and running losses, are incorporated^43,45^. To support the utilization of CTMs, total VOC emissions are mapped to SAPRC-07 species using sector-specific, domestically derived VOC profiles for different MS types^47^.

For non-exhaust particulate matter (PM) EFs, this study quantifies PM emissions from brake wear, tire wear, and road surface abrasion, while avoiding double-counting with road dust, which may include contributions from tailpipe emissions and soil resuspension^52^. The recommended EFs are differentiated by road type, including urban roads, rural roads, and highways. Measurements have shown that electric vehicles (EVs), due to their heavier weight, generate higher non-exhaust PM emissions than their fossil fuel counterparts^54^. Accordingly, the PM wear EFs for EVs are adjusted upward in this study by 8.3 ± 0.1% for tire wear and 12.5 ± 1.8% for brake wear.

For NH_3_ and CO_2_ EFs, measured NH_3_ EFs for light-duty gasoline passenger vehicles and heavy-duty diesel trucks are directly adopted^42^. For vehicle types lacking direct measurements, NH_3_ emissions are estimated using fuel-based scaling factors^42^. Fuel consumption data were collected from both literature^55^ and public web platforms, including the China Automotive Energy Consumption Query Platform (https://yhgscx.miit.gov.cn/fuel-consumption-web/mainPage), covering approximately 1,800 records. Relevant data can be retrieved using the Chinese keywords for “Passenger Car M1” and “Light-Duty Truck N1”. CO_2_ emissions are calculated using energy consumption data, net calorific values of fuels, CO_2_ emission factors, and carbon oxidation rates, as described in Eq. 4.

### Integration of non-road MS activity data

**Fig. 3:**
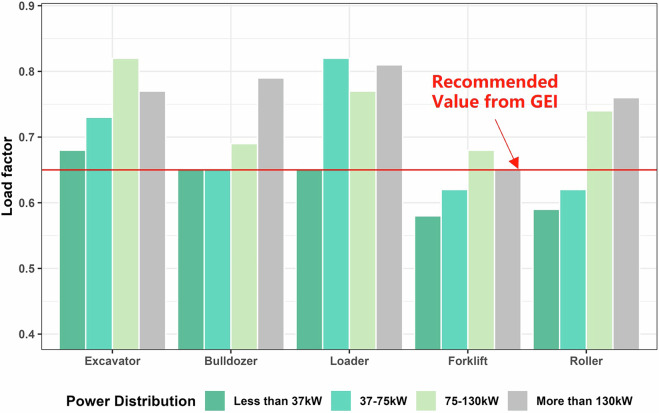
Comparisons of measured LF with the recommended values from GEI.

The activity data for non-road MS was mainly collected from GEI for non-road MS^23,27^. Detailed ownership data for various subcategories were collected from multiple official yearbooks: NRAM data, including provincial power distributions, were sourced from the China Agricultural Machinery Industry Yearbook (https://www.jgcm.ac.cn/ebook/zgnyjxgynj/2021/mobile/index.html#p=202); NRCM data came from the China Construction Machinery Industry Yearbook (https://cnki.nbsti.net/CSYDMirror/trade/Yearbook/Single/N2021040193?z=Z012). Fuel consumption data of RE were extracted from the China Railway Yearbook^56^, while the number of landing and take-off (LTO) cycles for civil aviation aircraft was estimated using the Civil Aviation Airport Production Statistics Bulletin (https://www.gov.cn/xinwen/2021-06/11/5617003/files/c51af61cc760406e82403d99d898f616.pdf). In addition to these official sources, measured data from published literature were also integrated into GMED, including EFs, LFs, and Ahr for NRM, as well as fuel consumption data for RE^21,22,57^. Moreover, the incorporation of measured Ahr data enabled NRAM emissions to better capture their seasonal operational characteristics. As shown in Fig. 3, comparisons between measured and GEI-recommended LFs indicate relative differences ranging from –10% to 26%, highlighting the impact of using regionally representative activity parameters.

### Spatiotemporal allocation of emissions in GMED

Vehicular emissions were first estimated by road grade at the county level and subsequently allocated to a 36 km resolution grid using county-level road density. To account for emissions from vehicles operating outside their registered cities, the spatial distribution methodology proposed by Zheng *et al*.^58^ was adopted. In this approach, vehicle emissions are first split into two parts based on statistical estimates of intra-city and inter-city VKT for each vehicle category. The emissions attributed to inter-city activity are then redistributed across all cities within the same province, using the share of each city’s road network length in the provincial total as a weighting factor. Moreover, the impact of COVID-19 on vehicle activity was incorporated based on variations in traffic flow derived from Intelligent Transportation System data^59^.

For NRM emissions, previous studies have primarily relied on proxies such as total NRAM power, GDP, or population for spatial allocation. However, these indicators may not sufficiently capture the actual operational characteristics of NRM. For example, NRAM emissions are more closely associated with cultivated land, rather than residential areas, while NRCM emissions are linked to construction activities, not total GDP which also includes service sectors. To improve spatial accuracy, this study employed high-resolution (30 m) land-use data from 2011 to 2020 (https://essd.copernicus.org/articles/13/3907/2021/)^60^. Cultivated land area was used to allocate NRAM emissions, under the assumption that agricultural machinery primarily operates in such areas (Eq. 23). For NRCM emissions, annual increases in impervious surfaces were used as a proxy, assuming construction-related emissions are concentrated in newly urbanized zones (Eq. 24). Emissions from RE and aircraft LTO cycles were allocated based on the spatial distribution of national railway mileage and airport locations, respectively.

23 $${E}_{{id},y,{\rm{prov}}}={E}_{y,{\rm{prov}}}\times \frac{{N}_{{id},y,{\rm{prov}}}}{{\sum }_{n}^{1}{N}_{{id},y,{\rm{prov}}}}$$

24 $${E}_{{id},y}={E}_{y}\times \frac{{N}_{{id},y}-{N}_{{id},y-1}}{{\sum }_{n}^{1}\left({N}_{{id},y}-{N}_{{id},y-1}\right)}$$

where *id* represents the index of a grid cell in the 36 km gridded emission inventory; *y* denotes the inventory year; prov indicates the Provinces in China; E_*id,y*,prov_ refers to the emissions allocated to grid *id* in year *y* and province *prov*; E_*y*,prov_ stands for the total NRAM emissions in year *y* and Province *prov*; For grids overlapping multiple provinces, emissions are averaged based on the number of overlapping regions. In Eq. 23, n represents the number of grids within a specific province, whereas in Eq. 24, n refers to the total number of 36 km grids across the whole country; N_*id*,*y*,prov_ indicates the number of sub-grid (i.e., cultivated land patches) within grid *id*; E_*id,y*_ refers to the emissions allocated to grid *id* in year *y*; E_*y*_ stands for the total NRCM emissions in year *y*; *N*_*id,y*_ *- N*_*id,y*-1_ in Eq. 24 represents the relative increase in impervious surface area within grid *id* from year *y* – 1 to *y*, and is used as a proxy for estimating changes in NRCM emissions.

**Fig. 4:**
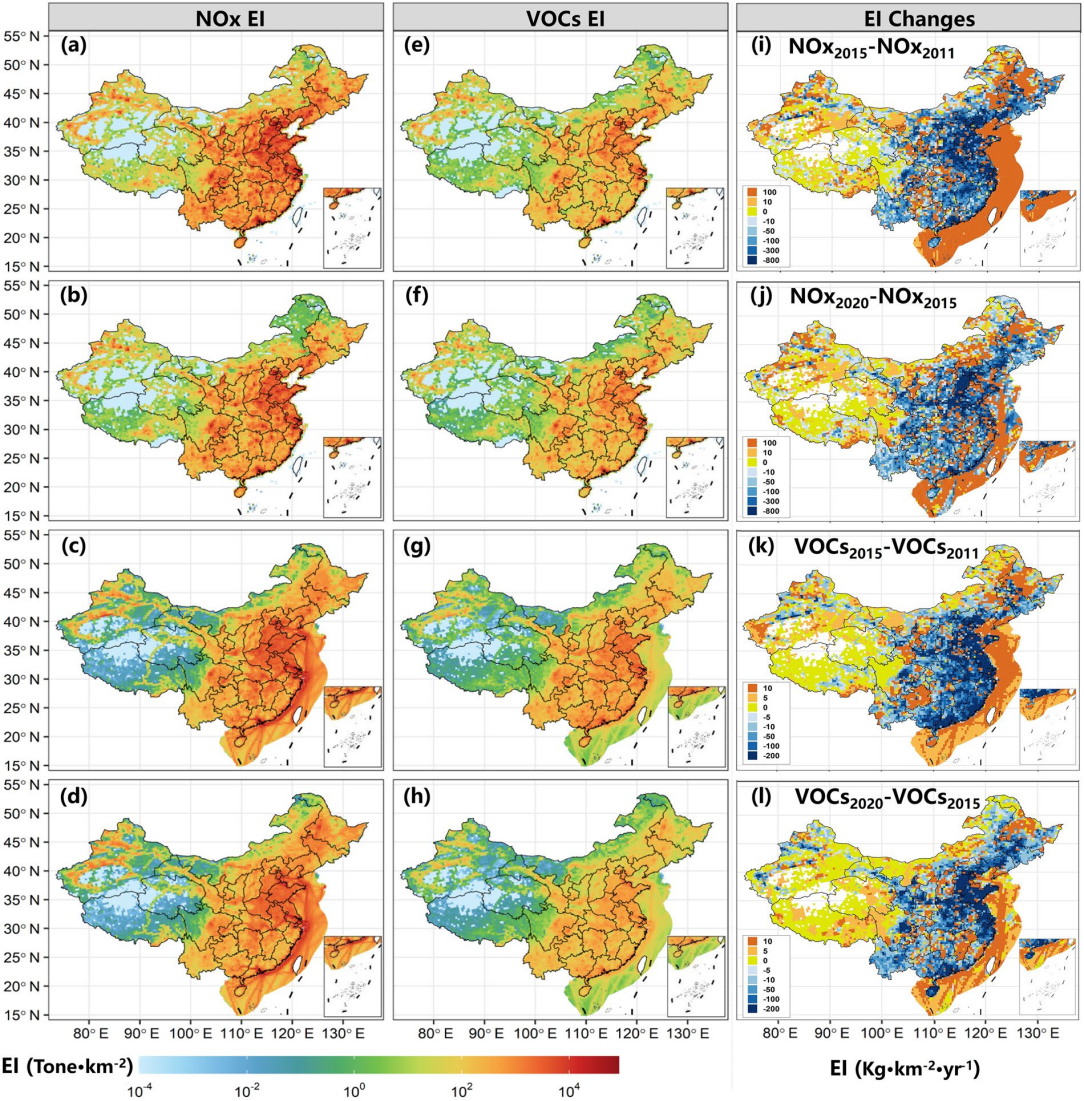
Emission intensity (EI) of gridded mobile source emission inventory. (**a**,**b**) NOx EI from On-road MS during 2011 (**a**) and 2020 (**b**); (**c,d**) NOx EI from Non-road MS during 2011 (**c**) and 2020 (**d**); (**e,f**) VOCs EI from On-road MS during 2011 (**e**) and 2020 (**f**); (**g,h**) VOCs EI from Non-road MS during 2011 (**g**) and 2020 (**h**) (**i**) NOx EI changes from MS between 2015 and 2011; (**j**) NOx EI changes from MS between 2020 and 2015; (**h**) VOCs EI changes from MS between 2015 and 2011; (**l**) VOCs EI changes from MS between 2020 and 2015; In (**i–l**), positive values represent an increase of EI, while negative values represent a decrease of EI.

Figure 4 illustrates the spatial distribution and temporal changes in emission intensity (EI) of NOx and VOCs from both on-road and non-road MS. Panels (a–d) show the NOx EI in 2011 and 2020, while panels (e–h) display the VOCs EI for the same years. Panels (i–l) present the EI changes between 2011–2015 and 2015–2020. The results reveal distinct spatial patterns and temporal trends in emission intensities, reflecting the impact of control measures and regional differences in emission characteristics of MS. In addition, monthly vehicular emissions were distributed using provincial-level highway freight and passenger turnover data to allocate VKT across months. Monthly emissions from NRAM and NRCM sources were estimated using the temporal variation in agricultural ammonia emissions and monthly housing completion area, respectively^22^. Emissions from other sources were assumed to follow a uniform monthly distribution.

### Uncertainty analysis

A Monte Carlo simulation with 10,000 iterations was conducted to evaluate the uncertainties in MS emissions from GMED. The uncertainties originated from several key parameters, including EF, VP, VKT, Ahr, average engine power, and LF. The coefficient of variation (CV), defined as the ratio of standard deviation to the mean, was used to quantify the variability of these parameters. The probability distributions and CV values were determined based on existing literature^16,21,32,42,61,62^. Ownership data for various MS types, obtained from official statistical yearbooks, were associated with low uncertainty. Accordingly, normal distributions were assigned to the VP of on-road and non-road MS, with CVs of 5% and 10%, respectively. VKT data, derived from surveys and empirical models, were also modelled using a normal distribution with a CV of 30%. Similarly, Ahr and LF followed normal distributions with CVs of 30%. Based on previous studies, lognormal distributions were applied to all types of EFs^16^. For on-road vehicle EFs, the CVs were set as follows: 17% for CO and NOx, 34% for PM_10_, PM_2.5_, SVOCs, and xLVOCs, 48% for HC, VOCs, and IVOCs, and 38% for NH_3_. For non-road MS, the CVs were 57% for CO, 42% for NOx, 6% for CO_2_, 40% for HC, and 47% for PM_2.5_. The CVs of VOCs and IVOCs were assumed to be the same as those of HC, while the CVs of PM_10_, SVOCs, and xLVOCs were assumed to match those of PM_2.5_.

## Data Records

The model-ready, gridded mobile source emission inventory developed by GMED is available at Zenodo^63^ 10.5281/zenodo.15262011. The dataset covers the years 2011 to 2020 and provides monthly emission estimates for a wide range of pollutants across various MS categories. The data are shared in compressed .zip format, with one *.zip* file for each MS category. Inside each archive, individual *.csv* files are provided, with each file corresponding to a specific year and month. The file naming convention is: *Year_Month_(MS type).csv*. A separate supplementary file (*Species_Description.csv*) lists the full names, units and classifications of pollutants included in the dataset.

**Fig. 5:**
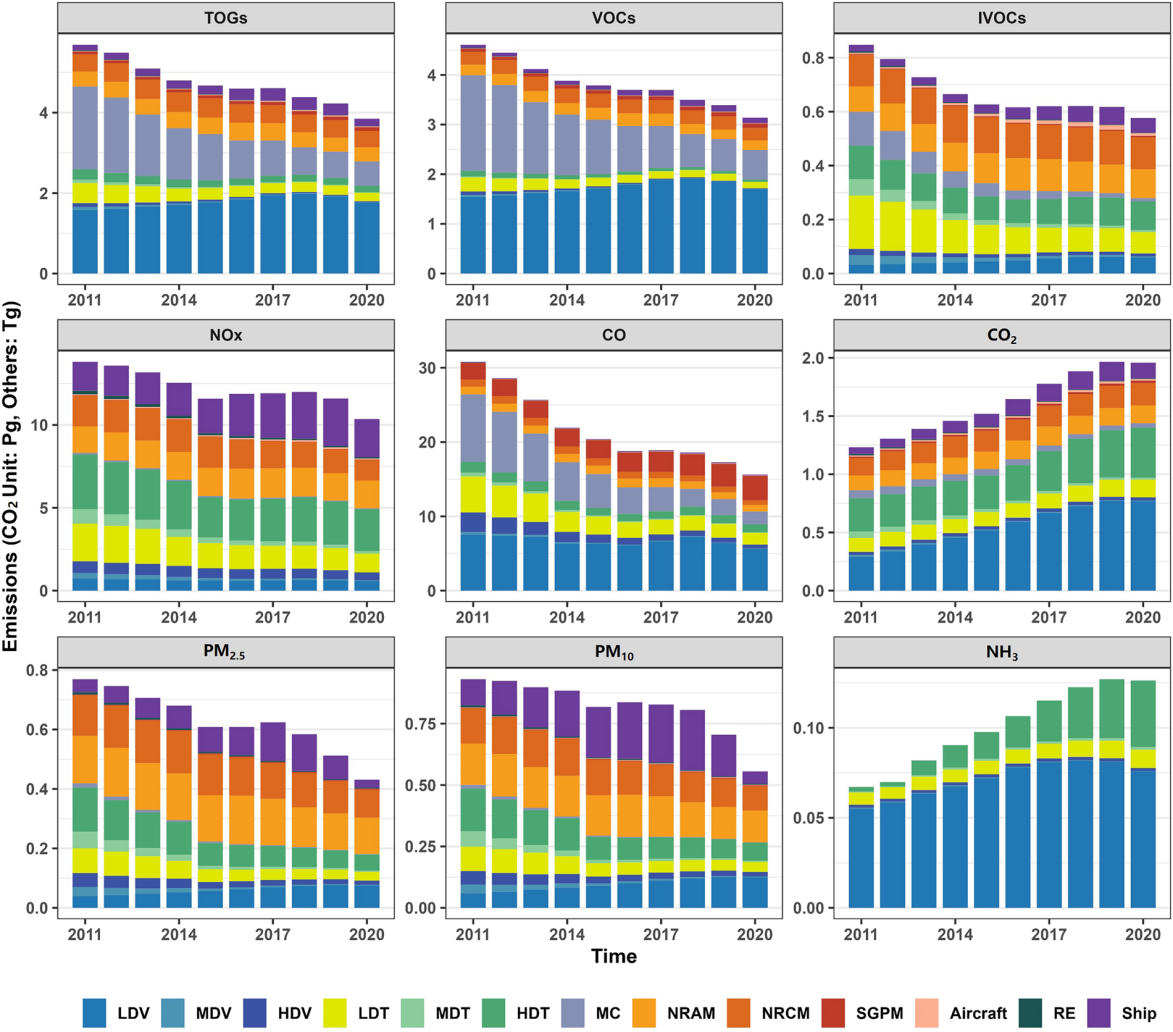
Trends in major pollutant emissions in GMED from 2011 to 2020. LDV: Light-duty passenger vehicle; MDV: Medium-duty passenger vehicle; HDV: Heavy-duty passenger vehicle; LDT: Light-duty truck; MDT: Medium-duty truck; HDT: Heavy-duty Truck; MC: Motorcycle; NRCM: Non-Road construction machinery; NRAM: Non-road agricultural machinery; SGPM: Small general-purpose machinery; RE: Railway internal combustion engines; TOGs: Total organic compounds (total amount of VOCs, IVOCs, SVOCs and xLVOCs).

**Fig. 6:**
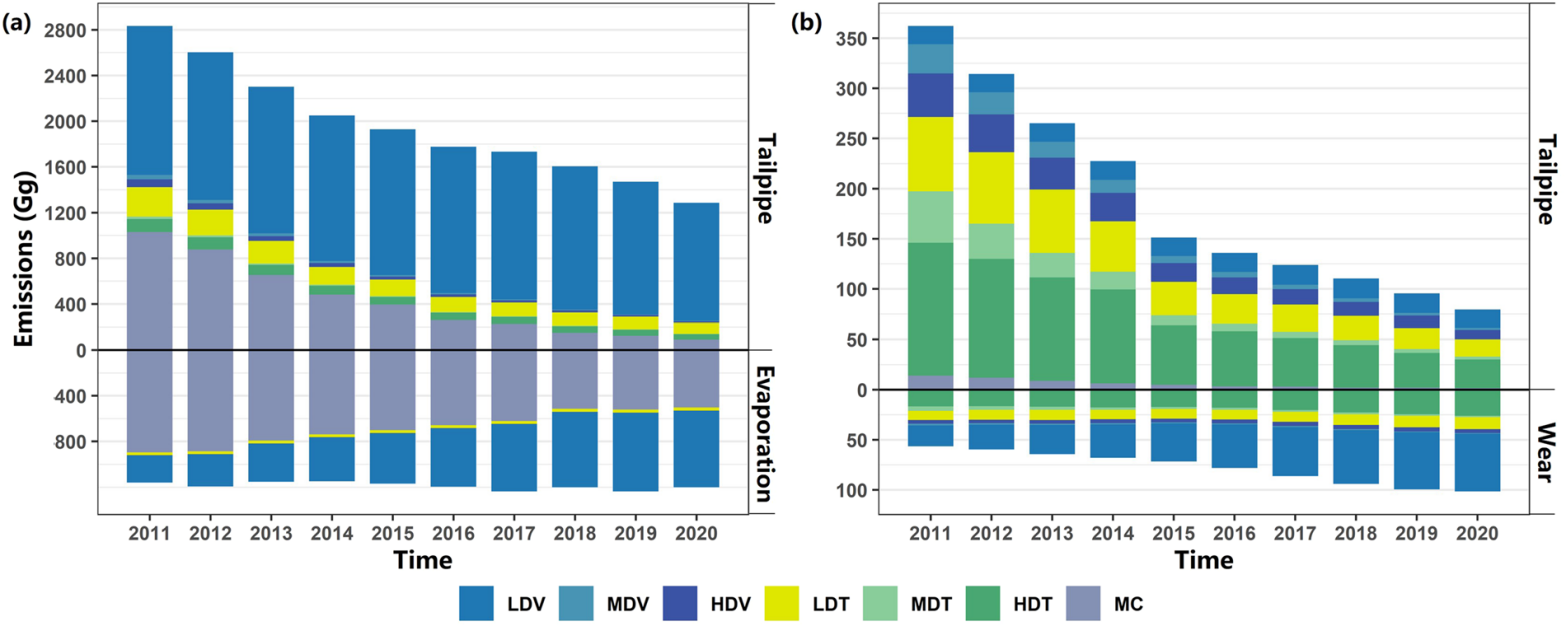
Emissions of different vehicle emission process. (**a**) VOCs; (**b**) PM_2.5_; The upper half of each subfigure depicts tailpipe emissions, whereas the lower half illustrates non-exhaust emissions; LDV: Light-duty passenger vehicle; MDV: Medium-duty passenger vehicle; HDV: Heavy-duty passenger vehicle; LDT: Light-duty truck; MDT: Medium-duty truck; HDT: Heavy-duty Truck; MC: Motorcycle. The wear and tear emissions of motorcycles were not considered in this study.

The MS categories included in the GMED are: (1) On-road vehicles (excluding motorcycles), including both tailpipe and non-exhaust emissions); (2) Motorcycles (saved separately), including evaporative VOC emissions; (3) Non-road construction machinery; (4) Non-road agricultural machinery; (5) Aircraft, including only emission during LTO cycles; (6) Ships, emissions within 200 nautical miles offshore are provided; (7) Small general-purpose machinery; (8) Railway internal combustion engines. The overall emission trends of different MS categories from 2011 to 2020 are illustrated in Fig. 5, while Fig. 6 presents the relative contributions of various emission processes to VOCs and PM_2.5_ emissions from vehicles.

Each .*csv* file contains two kinds of columns: (I)”FID_Grid” refers to the spatial grid identifier (see below); (II) Pollutant columns stands for the pollutant species included in the inventory. Each row in the file corresponds to the total monthly emissions for a specific grid cell. Emissions of VOCs are speciated into model-ready species based on the SAPRC-07 mechanism, reported in million moles per month. Less volatile organics (IVOCs, SVOCs, ELVOCs) are allocated into volatility bin sets based on their saturation vapor pressures, and reported in tonnes per month. Other gaseous and particulate pollutants are also reported in tonnes per month.

The spatial resolution of the GMED is 36 km × 36 km, and only grid cells with non-zero emissions are included in each file to reduce file size. In addition, due to varying VOC speciation rules across MS categories^47^, only the non-zero species are included in each .csv file. To support spatial mapping and integration with CTMs, a shapefile named “GMED_FID_Grid_36km.shp” is provided. This file defines the gridded domain used for the emission inventory and includes: (1) FID_Grid: Unique identifier used to match emission records; (2) Geographic information of each grid.

Trends of major pollutants emissions in GMED.

## Technical Validation

### Verification of simulated county-level vehicle population

**Fig. 7:**
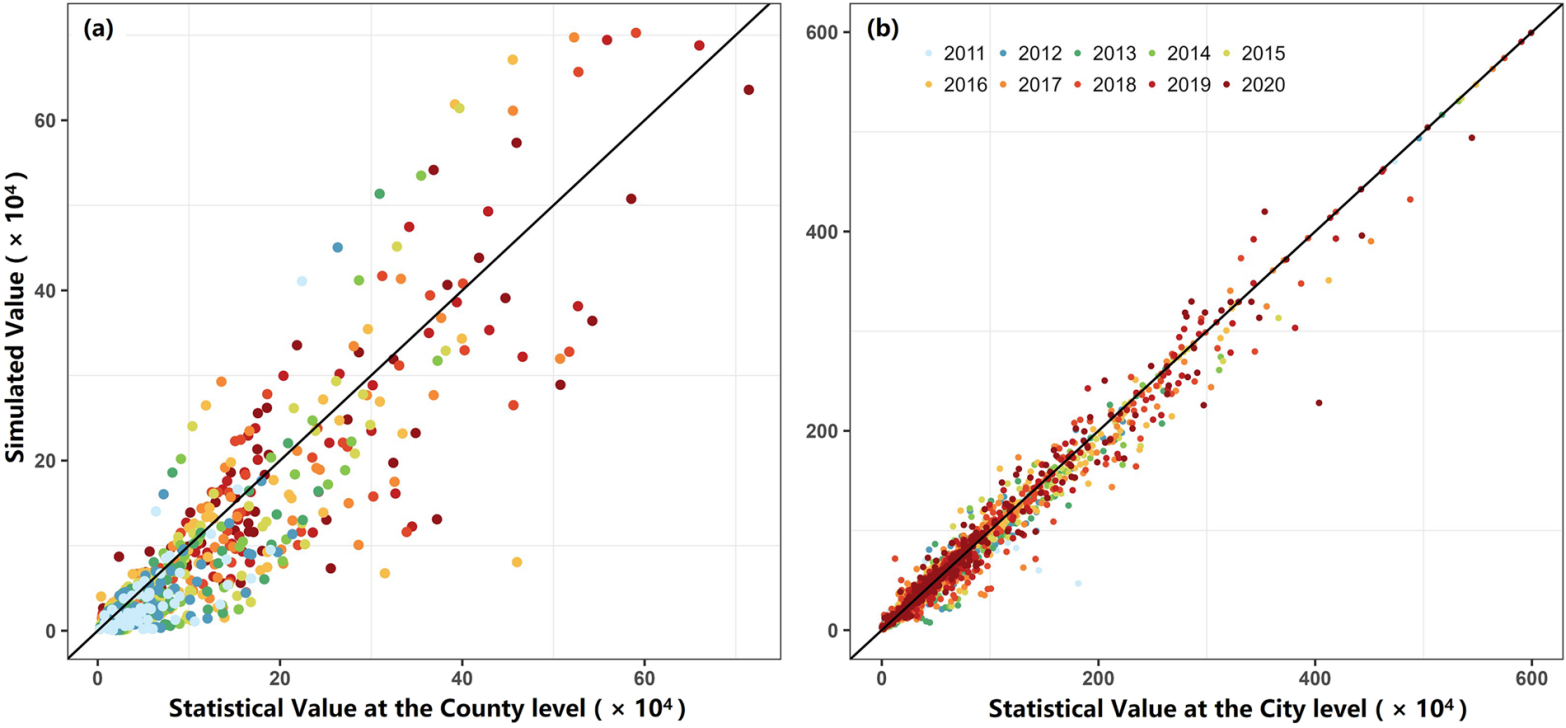
Verification of the VP spatial allocation results. (**a**) Comparison of simulated county-level VP and the corresponding statistical data. (**b**) Comparison of the simulated and statistical VP for each city.

Figure 7 compares the simulated county-level VP with statistical data, with the resulting *R*^2^ of 0.77 (Fig. 7a). Due to limited availability of county-level statistics, we further aggregated simulated county-level VPs to the city level and compared them with city-level statistical data (Fig. 7b). The average *R*^2^ across years exceeded 0.93. Specifically, 85% of cities showed *R*^2^ values above 0.6, while lower correlations were mainly observed in provinces such as Inner Mongolia, Jilin, Liaoning, Qinghai, Heilongjiang, and Guangxi. Despite these regional discrepancies, the simulation results are generally consistent with the available statistics.

### Validation of ES distributions

**Fig. 8:**
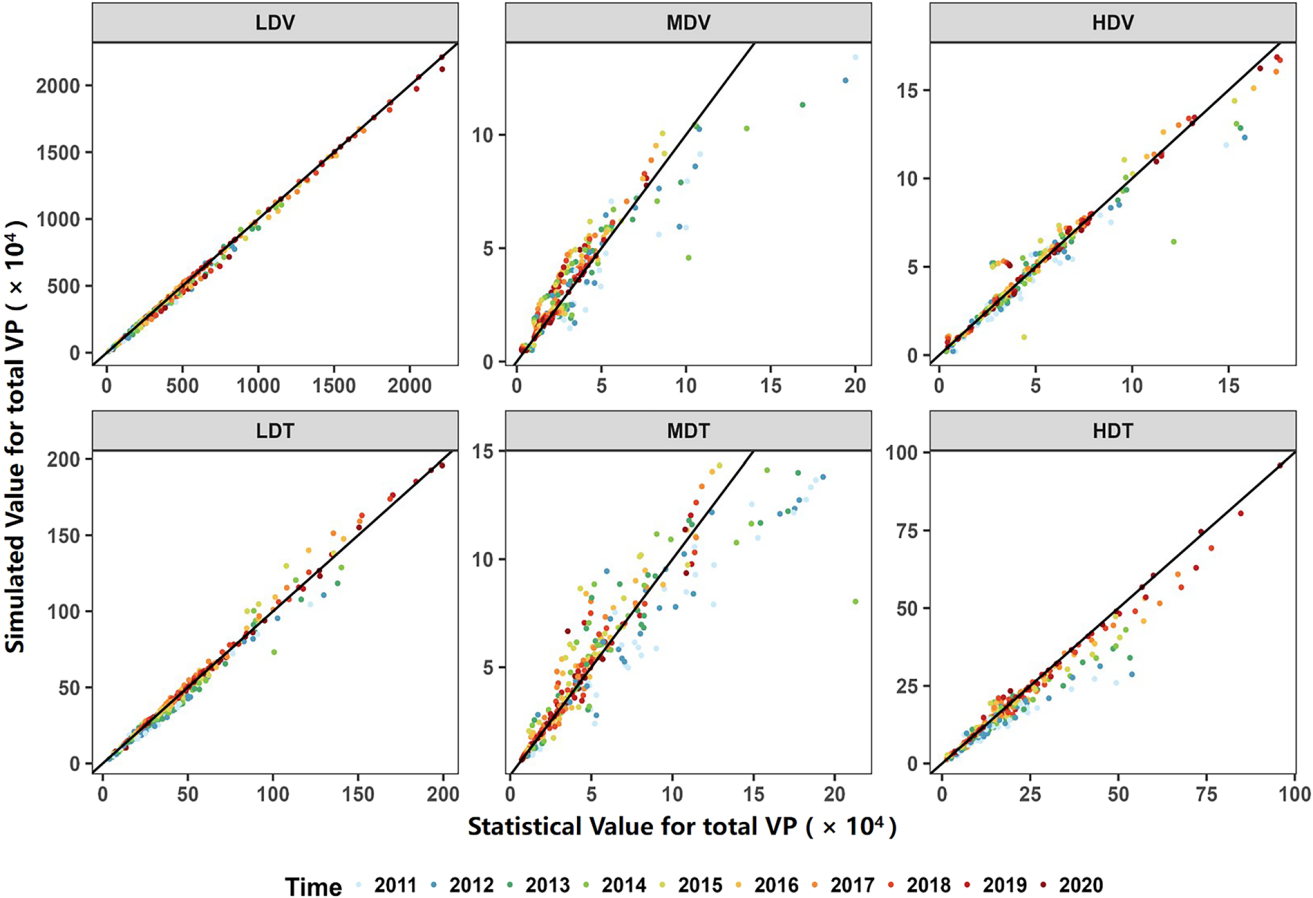
Validation of ES distribution. Comparison of statistical total VP and simulated values; LDV: Light-duty passenger vehicle; MDV: Medium-duty passenger vehicle; HDV: Heavy-duty passenger vehicle; LDT: Light-duty truck; MDT: Medium-duty truck; HDT: Heavy-duty Truck.

To check whether the simulated ES and age distributions are reliable, we validated them using the improved survival rate function. According to its fundamental assumption, if any two of the three variables (survival rate, newly registered VP, and total VP) are known, the third can be estimated. Using the improved survival rate function and data on newly registered vehicles, we simulated the total VP and compared it against statistical records. As shown in Fig. 8, the simulated results closely matched statistical values for light-duty and heavy-duty vehicles. In contrast, medium-duty vehicles exhibited lower accuracy; however, their limited share in the total fleet implies minimal impact on aggregate emissions. Compared with existing approaches, the use of the refined survival rate function and more detailed vehicle classification improved the correlation between simulated and statistical VP from 0.84 (Current parameters from literatures^30^) to 0.92, representing an enhancement of approximately 10%. Furthermore, the resulting ES distributions align with regional emission control practices. For instance, as illustrated in Fig. S3, due to more stringent and timely emission regulations, Beijing exhibits a higher proportion of cleaner vehicles (i.e., newer ES) compared to Jiangxi Province, which more closely reflects the national average.

### Comparison with other studies and uncertainty analysis

**Fig. 9:**
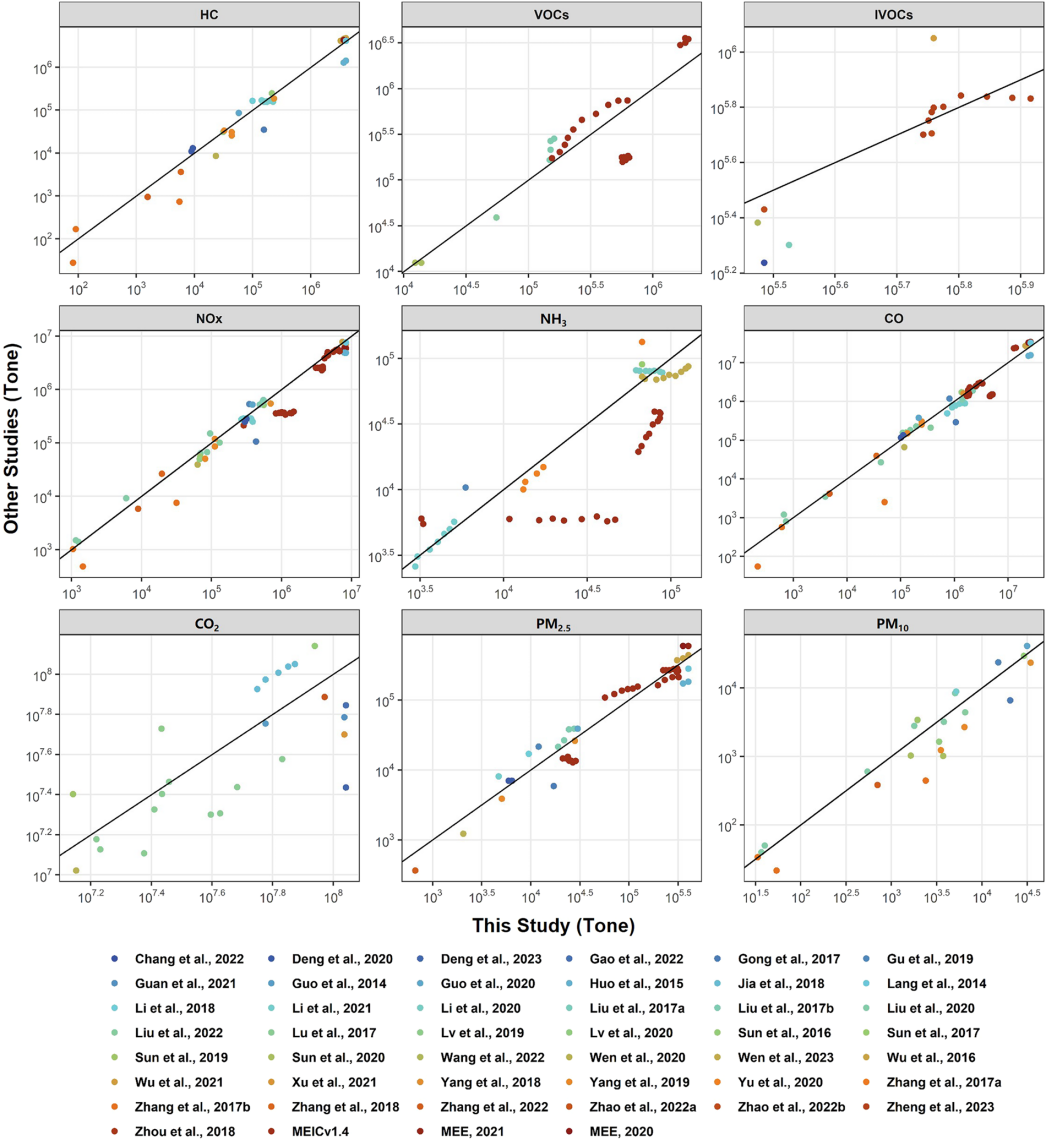
Validation of the estimated emissions with other researches, encompassing various pollutants, MS and regions. MEIC: Multi-resolution Emission Inventory for China; MEE: China Mobile Source Environmental Management Annual Report.

This study provides a comprehensive evaluation of GMED using data from 46 studies^3,16,22,30,32,33,41,42,48,50,61,64–98^ (Fig. 9). Emissions were compared at both national and provincial levels, incorporating data from a range of MS, including on-road vehicles, NRAM, NRCM, aircraft, and RE. Overall, the estimated emissions are broadly consistent with existing inventories. For each literature dataset, we extracted GMED estimates under comparable spatial, temporal, and sectoral conditions, and computed the mean absolute error (MAE) as the absolute difference between the two datasets, normalized by the reference value. The MAE for total MS emissions of CO, CO_2_, HC, VOCs, IVOCs, NOx, NH_3_, PM_2.5_, and PM_10_, when compared to previous studies, are 32%, 47%, 33%, 68%, 20%, 28%, 33%, 41%, and 54%, respectively. Although discrepancies exist across different studies, most are within the same order of magnitude. These differences mainly arise from variations in emission correction methods, fleet composition, and EFs. For instance, our previous study estimated 2017 IVOC emissions at 5.075 million tons, lower than the 5.701 million tons reported in this study. Although both studies used the same IVOCs EFs, but differences in the ES allocation, VKT, LF, and fuel compositions contribute to the observed discrepancies^22^. This study also compares the emission results from the China Mobile Source Environmental Management Annual Report^3,65^, with resulting MAE values for CO, NOx, and HC emissions from on-road vehicles being 18%, 19%, and 8%, respectively. In addition, when compared to the MEIC v1.4 MS emissions, the MAE for HC, NOx, PM_2.5_, CO, and NH_3_ emissions from 2011 to 2020 are 61%, 22%, 15%, 47%, and 63%, respectively.

While the comparisons above demonstrate that GMED estimates are of the same order of magnitude as existing studies, the differences in results may stem from variations in accounting methodologies, updates to emission inventory models, and uncertainties inherent in the methods themselves. To further assess the reliability of GMED, this study incorporated an independent validation using high resolution emission inventories. Moreover, inventories based on real-time traffic flow have been shown to better capture the spatiotemporal variability of MS emissions. We compared GMED with three real-time inventories^33,73,81^, where the MAE values for HC, NOx, CO, PM_2.5_, and PM_10_ emissions were all below 60% (Fig. S4). Specifically, the average MAE values for HC and NO_x_ were 25% and 23%, indicating strong consistency with dynamic, high-resolution datasets.

In terms of uncertainty analysis through Monte Carlo simulations, it is worth to note that the current analysis follows commonly used methodologies from previous studies. However, the Monte Carlo simulations only account for part of key parameters (e.g., ownerships, EFs, VKT, Ahr, and LF), while overlooking other critical factors (e.g., proxy parameters for spatiotemporal allocation, meteorological conditions, deterioration factors, fuel composition, and operating conditions). These omitted factors could also affect the final estimated emissions but are difficult to quantify. As a result, the quantitative evaluation results of Monte Carlo simulations should be regarded as providing a certain reference value. Ultimately, the 95% confidence interval for the emission inventory results spans from −25% to 48%.

### Limitations and prospect

The uncertainty of multi-year emission inventories is influenced by a variety of factors, and validating inventory results has long remained a challenging issue, particularly when assessing emissions from individual sources. In this study, we conducted a comparison with peer-reviewed results. While the estimated emissions were found to be generally consistent with those of other studies, some discrepancies were observed. This study incorporated recently published real-world measurement data and a more comprehensive emission process, including wear-related emissions. These data, derived from measurements and surveys, significantly enhance the representativeness and reliability of the inventory results. However, the current inventory still requires further refinement. First, due to data availability limitations, this study did not account for interannual variations in fuel composition of MS. Second, the VKT correction algorithms based on vehicle age should also consider regional differences. Furthermore, the current EF correction models predominantly focus on conventional pollutants, such as PM and NOx. The development of EF correction models for unconventional pollutants, such as IVOCs, is also necessary. Finally, MS emissions that occur outside their registered locations, due to interregional transportation, need to be considered. In this study, we employed a simplified approach by reallocating emissions based on intercity and intra-city allocation factors and road network lengths^58^. Our dataset is primarily designed for regional-scale air quality modeling (e.g., province level) over annual to monthly timeframes. For high-resolution (e.g., street-level) simulations, we acknowledge that finer-scale, city-specific inventories may be more appropriate. Future work could use environmental big data, such as vehicle trajectories, to further optimize emissions allocation.

## Supplementary information

Supplementary Information of

## Data Availability

The code used for data visualization in this study is publicly available from 10.5281/zenodo.15262011. All scripts were developed in R (version 4.4.2).
