# Supplementary material for: Gridded mobile source emissions with multiple processes and pollutants from 2011 to 2020 in China
Source: Sci Data. 2025 Aug 1;12:1344. doi: 10.1038/s41597-025-05690-6 (PMC12316859; doi:10.1038/s41597-025-05690-6)
Supplement: Supplementary file 1 — Supplementary Information of [file 41597_2025_5690_MOESM1_ESM.pdf]

Supplement of

Gridded mobile source emissions with multiple processes and pollutants from 2011 to 2020 in China

Authors

Junchao Zhao[1,2], Zhaofeng Lv[1], Zhenyu Luo[1], Zhining Zhang[1], Haitong Zhe Sun[3], Wenxin Cao[1], Wen Yi[1], Yongyue Wang[1], Hezhong Tian[4], Yan Ding[2]*, Kebin He[1], Huan Liu[1]*

Affiliations

1. State Key Laboratory of Regional Environment and Sustainability, School of Environment, Tsinghua University, Beijing 100084, China
2. Key Laboratory of Vehicle Emission Control and Simulation of Ministry of Ecology and Environment, Vehicle Emission Control Center, Chinese Research Academy of Environmental Sciences
3. Centre for Sustainable Medicine (CoSM), National University of Singapore, Singapore, 119228, Singapore
4. State Key Joint Laboratory of Environmental Simulation & Pollution Control, School of Environment, Beijing Normal University, Beijing 100875, China

Corresponding author: Huan Liu (liu_env@tsinghua.edu.cn); Yan Ding (dingyan@craes.org.cn).

Number of figures: 4; Number of tables: 3.

Supplementary Figures

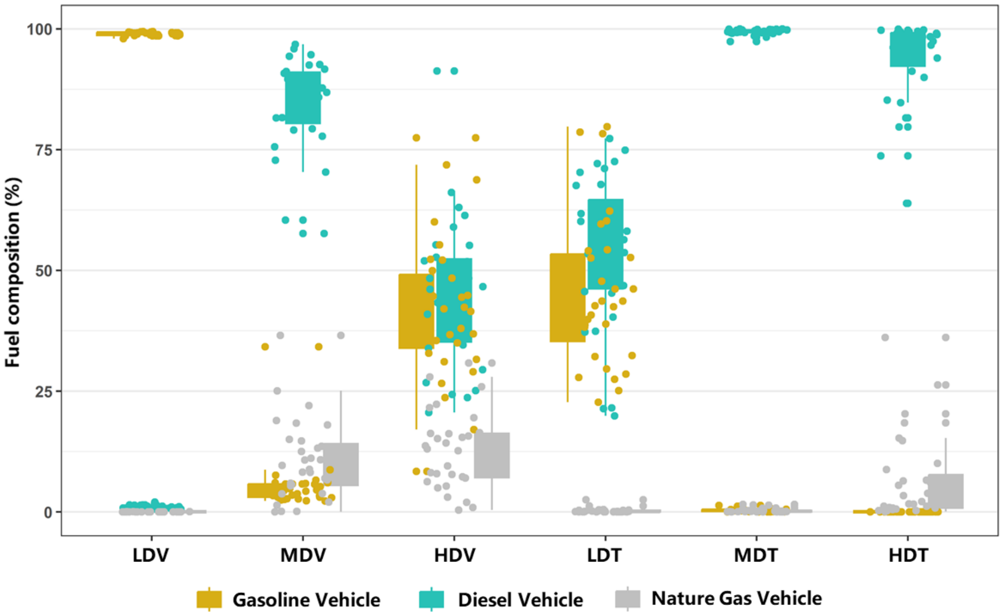

Fig. S1. Distribution of the fuel composition across different vehicle categories in each province; The range of variation depicted by the box plot highlights disparities in fuel composition among provinces, while the scatter plot provides specific proportion values for each province; LDV: Light-duty passenger vehicle; MDV: Medium-duty passenger vehicle; HDV: Heavy-duty passenger vehicle; LDT: Light-duty truck; MDT: Medium-duty truck; HDT: Heavy-duty Truck.

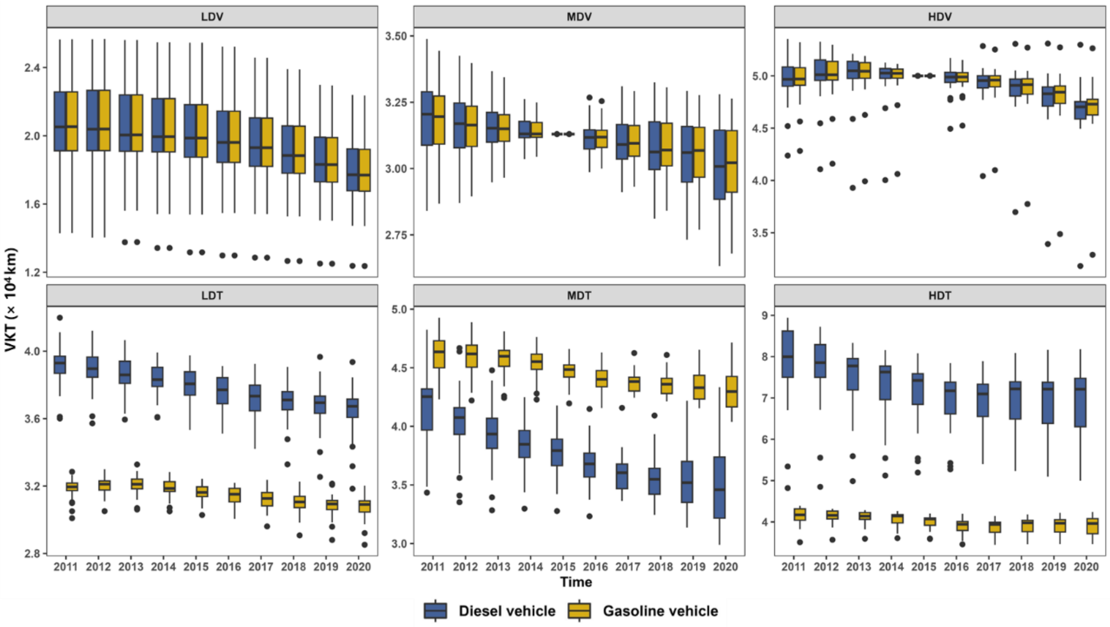

Fig. S2. Multi-year distribution of vehicle kilometers traveled (VKT) for various vehicle categories. For trucks (LDT, MDT, HDT), the box plots represent the variability in VKT across different vehicle ages. For passenger vehicles (LDV, MDV, HDV), the box plots illustrate the provincial variability in VKT; LDV: Light-duty passenger vehicle; MDV: Medium-duty passenger vehicle; HDV: Heavy-duty passenger vehicle; LDT: Light-duty truck; MDT: Medium-duty truck; HDT: Heavy-duty Truck.

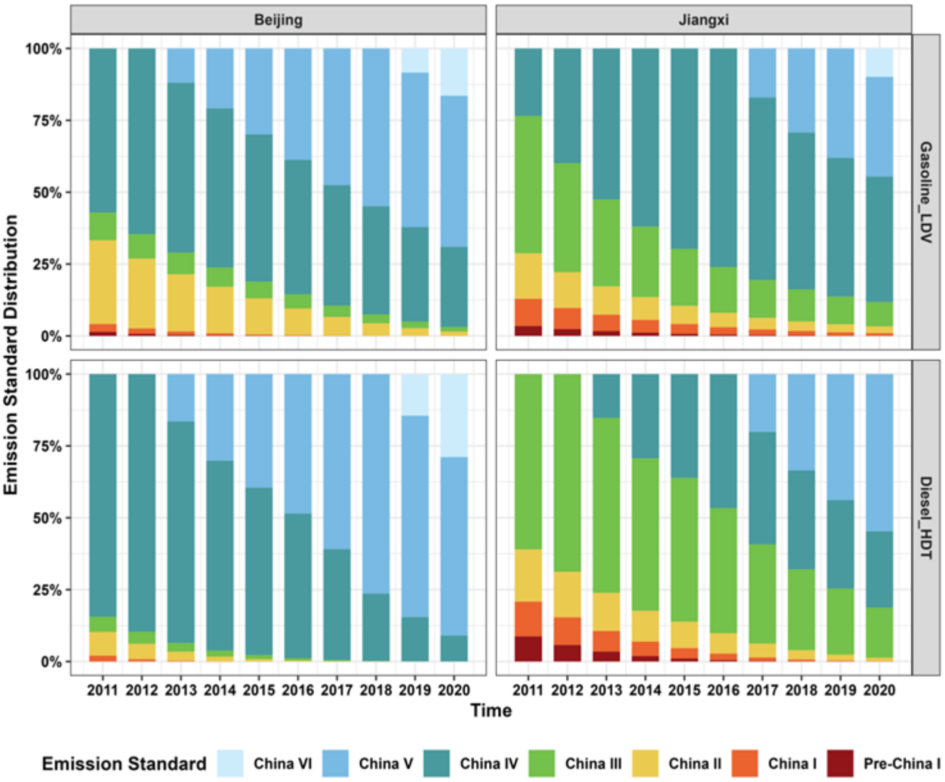

Fig. S3 Detailed emission standard distribution, using Beijing and Jiangxi Province as examples.

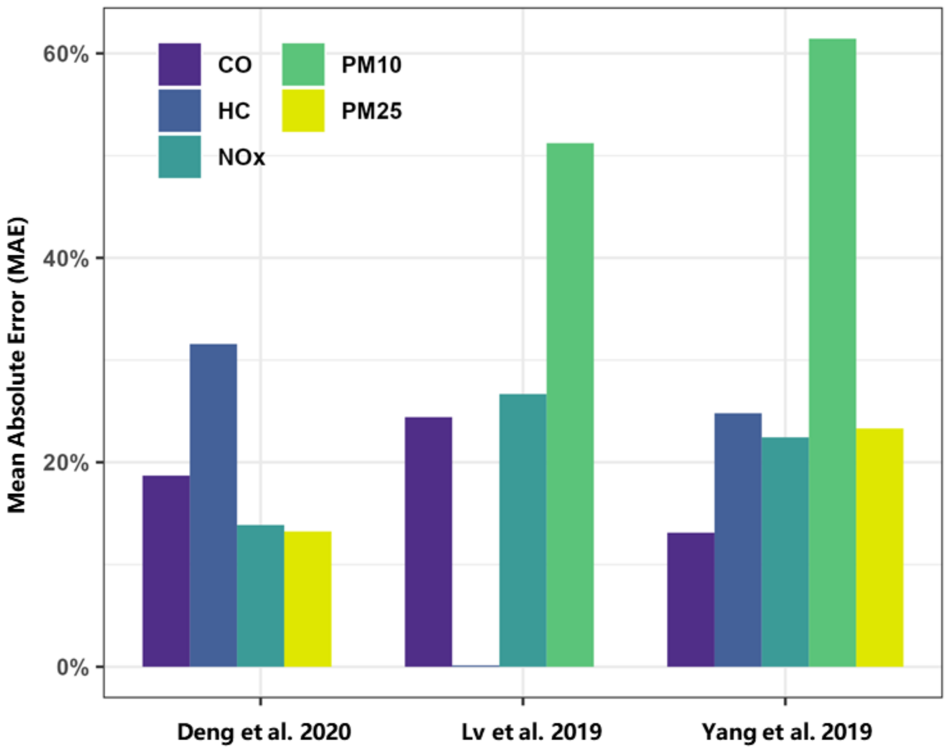

Fig. S4 Validation of the estimated emissions with three high resolution emission inventories.

Supplementary Tables

Table S1. Key parameters of the improved survival curve functions

Provinces	LDT		MDT		HDT		LDV		MDV		HDV	
	T	b	T	b	T	b	T	b	T	b	T	b
Anhui	17	5	26.5	15	16	5	24.5	7	21	7	15.5	5
Beijing	16.5	5	24.5	15	13	5	16.5	5	24.5	10	21	8
Fujian	17	5	25	15	16	5	26	5	25	10.5	14	5
Gansu	19	5	17	5	21	5	35	7.5	19	6.5	15.5	5
Guangdong	18	5	21	8	19.5	8.5	23.5	6	18.5	5.5	14.5	5
Guangxi	20	5	17.5	5	25	5	35	8	21.5	6	16.5	5
Guizhou	17	5.5	25	15	16	5	29.5	15	23.5	14.5	15.5	5
Hainan	18	5	19	7	16.5	5	35	8.5	16	5	15	5
Hebei	27.5	14.5	25	15	18	9.5	32.5	12	27.5	15	17.5	5
Henan	20	8.5	24.5	15	17.5	8	35	15	16	5.5	15	5
Heilongjiang	22	5	20	5	35	13.5	35	7.5	27.5	13.5	19	6.5
Hubei	17	5	26	15	18.5	5	26.5	5	25.5	10	16	5
Hunan	17	5	16	5	20	5	32.5	14.5	16	5	15	5
Jilin	19	5	21	9	22.5	5	35	8	29.5	14	16.5	5
Jiangsu	16	5	15	5	20	8.5	25	11	27	15	15.5	5
Jiangxi	17	5	16.5	6	16.5	5	24	5.5	14.5	5	15.5	5
Liaoning	23	5	28.5	15	35	14.5	35	7	30	10.5	28.5	14.5
Inner Mongolia	21.5	5	18	5	23.5	5	35	7.5	28	11	25	11.5
Ningxia	19.5	5	21.5	9.5	24	5	35	8	27.5	12	20	8
Qinghai	18.5	5.5	18.5	5.5	22	5	35	7.5	30	15	18.5	5.5
Shandong	19	5	16	5	18	6	35	8	28	15	18	5.5
Shanxi	16	5	15.5	6	12.5	5	29.5	10.5	29.5	15	23	6.5
Shaanxi	15.5	5	23.5	15	15.5	7	22.5	6	14.5	5	20	10
Shanghai	13.5	5	23.5	9	27.5	14.5	17	5.5	16	5	24.5	15
Sichuan	16.5	5	15	5	16.5	5	21.5	5	15	5	15.5	5
Tianjin	16.5	5	15	5	15.5	5	18	5	20.5	7.5	15.5	5
Tibet	35	9	34.5	15	26	5	35	7.5	28	15	20	15
Xinjiang	19.5	5	19	5	30.5	6	35	7.5	20	5	20.5	7.5
Yunnan	17.5	5	25.5	15	18.5	5	31	14.5	15.5	5	19	7
Zhejiang	18	5.5	15.5	5	20	9.5	33	14.5	17	5.5	17	6
Chongqing	15.5	5	14.5	5	16	6	19	5.5	15.5	5	16	5

Table S2 Empirical functions of age-VKT curve for passenger vehicles

Vehicle types	Fuel types	Age-VKT curve $f(M)$[1]
LDV	Gasoline vehicle	VKT = −6659.44×ln(M)+26818.24
	Diesel vehicle	VKT = −6412.37×ln(M)+25989.16
MPV/HPV	Gasoline vehicle	VKT = −13266.46×ln(M)+54676.71
	Diesel vehicle	VKT = −17677.87×ln(M)+68994.05

Table S3 The composition of mobile source (MS) EF database in GMED

Pollutants	MS categories	Notes	References
CO, HC, NOx, $PM_{2.5}$, and PM_{10}	Vehicle	Emission Standards (ES) from pre-China I to China V	GEI for on-road MS[2]
CO, HC, NOx, $PM_{2.5}$, and PM_{10}	Non-road MS	ES from pre-China I to China III (only for NRM)	GEI for non-road MS[3]
CO, HC, NOx, $PM_{2.5}$, and PM_{10}	Vehicle	ES: China VI	Zhang et al.[4]; Yang et al.[5]; Prati and Costagliola[6]; Li et al.[7]; Gómez et al.[8],
$PM_{2.5}$, and PM_{10}	Vehicle wear emission	Classified by different roads	Ntziachristos and Boulter[9]
VOCs	Vehicle tailpipe emission	ES from pre-China I to China VI	GEI for VOCs[10]; Zhang et al.[11]
VOCs	Vehicle evaporative emission	ES from pre-China I to China VI	Liu et al.[12]; Man et al.[13]
IVOCs, SVOCs, and xLVOCs	Vehicle	ES from pre-China I to China VI	Qi et al.[14]; Zhao et al.[15]; Liu et al.[16]; Wang et al.[17]
NH_3	Vehicle	Classified by different types	Wen et al.[18]
Fuel consumption	Vehicle	For CO_2 calculation	China Automotive Energy Consumption Query Platform (https://yhgscx.miit.gov.cn/fuel-consumption-web/mainPage)
Fuel consumption	Non-road MS	For CO_2 calculation	GEI for non-road MS[3]
CO, HC, NOx, $PM_{2.5}$, and PM_{10}	Vehicle	Correlation methods for Emission factor	COPERT model[19]<br>GEI for on-road MS[2]
NOx	Vehicle	Correlation methods for Emission factor	MOVES[20]
VOCs	All types of MS	Mapping to SAPRC07 species	Sha et al.[21]
IVOCs, SVOCs, and xLVOCs	All types of MS	Mapping to VBS bins	Chang et al.[22]; Qi et al.[23]; Zheng et al.[24]

REFERENCES

1. Sun, S. *et al.* Developing a vehicle emission inventory with high temporal-spatial resolution in Tianjin, China. *Sci. Total Environ.* 776, 145873 (2021) doi:10.1016/j.scitotenv.2021.145873.
2. MEE. *Ministry of Ecology and Environment of the People's Republic of China). Technical Guidelines for Compilation of Air Pollutant Emission Inventory of Road Vehicles*. (2014).
3. MEE. *Ministry of Ecology and Environment of the People's Republic of China). Technical Guidelines for Compilation of Non-Road Mobile Source Air Pollutant Emission*. (2014).
4. Zhang, M. *et al.* Effects of ethanol and aromatic compositions on regulated and unregulated emissions of E10-fuelled China-6 compliant gasoline direct injection vehicles. *Renew. Energy* 176, 322–333 (2021) doi:10.1016/j.renene.2021.03.029.
5. Yang, Z. *et al.* Real-world gaseous emission characteristics of Euro 6b light-duty gasoline- and diesel-fueled vehicles. *Transp. Res. Part Transp. Environ.* 78, 102215 (2020) doi:10.1016/j.trd.2019.102215.
6. Prati, M. V. & Costagliola, M. A. Real driving emissions of Euro 6 electric/gasoline hybrid and natural gas vehicles. *Transp. Res. Part Transp. Environ.* 113, 103509 (2022) doi:10.1016/j.trd.2022.103509.
7. Li, X. *et al.* Integrated effects of SCR, velocity, and Air-fuel Ratio on gaseous pollutants and CO2 emissions from China V and VI heavy-duty diesel vehicles. *Sci. Total Environ.* 811, 152311 (2022) doi:10.1016/j.scitotenv.2021.152311.
8. Gómez, A. *et al.* Comparison of real driving emissions from Euro VI buses with diesel and compressed natural gas fuels. *Fuel* 289, 119836 (2021) doi:10.1016/j.fuel.2020.119836.
9. Ntziachristos, L. & Boulter, P. *EMEP/EEA Air Pollutant Emissions Inventory Guidebook 2013: Road Vehicle Tyre and Brake Wear*. (2013).
10. MEE. *Ministry of Ecology and Environment of the People's Republic of China). Technical Guidelines for Compilation of Atmospheric Volatile Organic Compound*

Emission Inventory. (2014).

11. Zhang, Z. *et al.* Updating emission inventories for vehicular organic gases: Indications from cold-start and temperature effects on advanced technology cars. *Sci. Total Environ.* 882, 163544 (2023) doi:10.1016/j.scitotenv.2023.163544.
12. Liu, Y. *et al.* Evaporative emission from China 5 and China 6 gasoline vehicles: Emission factors, profiles and future perspective. *J. Clean. Prod.* 331, 129861 (2022) doi:10.1016/j.jclepro.2021.129861.
13. Man, H. *et al.* VOCs evaporative emissions from vehicles in China: Species characteristics of different emission processes. *Environ. Sci. Ecotechnology* 1, 100002 (2020) doi:10.1016/j.ese.2019.100002.
14. Qi, L. *et al.* Primary organic gas emissions from gasoline vehicles in China: Factors, composition and trends. *Environ. Pollut.* 290, 117984 (2021) doi:10.1016/j.envpol.2021.117984.
15. Zhao, J. *et al.* An updated comprehensive IVOC emission inventory for mobile sources in China. *Sci. Total Environ.* 851, 158312 (2022) doi:10.1016/j.scitotenv.2022.158312.
16. Liu, Y. *et al.* Identification of two main origins of intermediate-volatility organic compound emissions from vehicles in China through two-phase simultaneous characterization. *Environ. Pollut.* 281, 117020 (2021) doi:10.1016/j.envpol.2021.117020.
17. Wang, A. *et al.* Measurement-based intermediate volatility organic compound emission inventory from on-road vehicle exhaust in China. *Environ. Pollut.* 310, 119887 (2022) doi:10.1016/j.envpol.2022.119887.
18. Wen, Y., Zhang, S., Wu, Y. & Hao, J. Vehicular ammonia emissions: an underappreciated emission source in densely populated areas. *Atmospheric Chem. Phys.* 23, 3819–3828 (2023) doi:10.5194/acp-23-3819-2023.
19. Ntziachristos, L. & Samaras, Z. *COPERT III, Computer Programme to Calculate Emissions from Road Transport*. (2000).
20. USEPA. *Motor Vehicle Emission Simulator: MOVES5. Office of Transportation and Air Quality. US Environmental Protection Agency. Ann Arbor, MI. November 2024.*

(2024).

21. Sha, Q. *et al.* A newly integrated dataset of volatile organic compounds (VOCs) source profiles and implications for the future development of VOCs profiles in China. *Sci. Total Environ.* 793, 148348 (2021) doi:10.1016/j.scitotenv.2021.148348.
22. Chang, X. *et al.* Full-volatility emission framework corrects missing and underestimated secondary organic aerosol sources. *One Earth* 5, 403–412 (2022) doi:10.1016/j.oneear.2022.03.015.
23. Qi, L. *et al.* Intermediate-Volatility Organic Compound Emissions from Nonroad Construction Machinery under Different Operation Modes. *Environ. Sci. Technol.* 53, 13832–13840 (2019) doi:10.1021/acs.est.9b01316.
24. Zheng, H. *et al.* Trends of Full-Volatility Organic Emissions in China from 2005 to 2019 and Their Organic Aerosol Formation Potentials. *Environ. Sci. Technol. Lett.* 10, 137–144 (2023) doi:10.1021/acs.estlett.2c00944.
